# Evolution and phylogenetic distribution of *endo*-α-mannosidase

**DOI:** 10.1093/glycob/cwad041

**Published:** 2023-05-18

**Authors:** Łukasz F Sobala

**Affiliations:** Laboratory of Glycobiology, Hirszfeld Institute of Immunology and Experimental Therapy, Weigla 12, 53-114 Wroclaw, Poland

**Keywords:** endomannosidase, evolution, GH99, MANEA, protist

## Abstract

While glycans underlie many biological processes, such as protein folding, cell adhesion, and cell–cell recognition, deep evolution of glycosylation machinery remains an understudied topic. N-linked glycosylation is a conserved process in which mannosidases are key trimming enzymes. One of them is the glycoprotein *endo*-α-1,2-mannosidase which participates in the initial trimming of mannose moieties from an N-linked glycan inside the *cis*-Golgi. It is unique as the only endo-acting mannosidase found in this organelle. Relatively little is known about its origins and evolutionary history; so far it was reported to occur only in vertebrates. In this work, a taxon-rich bioinformatic survey to unravel the evolutionary history of this enzyme, including all major eukaryotic clades and a wide representation of animals, is presented. The endomannosidase was found to be more widely distributed in animals and other eukaryotes. The protein motif changes in context of the canonical animal enzyme were tracked. Additionally, the data show the two canonical vertebrate endomannosidase genes, *MANEA* and *MANEAL*, arose at the second round of the two vertebrate genome duplications and one more vertebrate paralog, *CMANEAL*, is uncovered. Finally, a framework where N-glycosylation co-evolved with complex multicellularity is described. A better understanding of the evolution of core glycosylation pathways is pivotal to understanding biology of eukaryotes in general, and the Golgi apparatus in particular. This systematic analysis of the endomannosidase evolution is one step toward this goal.

## Introduction

Glycans are integral to the structures of most biomolecules and participate in many processes that are instrumental to life. Protein N-glycosylation in the endoplasmic reticulum is one of the protein quality control and chaperoning pathways and depends on concerted activity of biosynthetic enzymes, lectins, and transporters. Oligosaccharyltransferase (OST)-based N-glycosylation is present in many eukaryotic species and some archaea (Kelleher and Gilmore 2006). The evolution of the OST complex, central to this process, has been studied in some detail already and detecting it in archaea was a finding which strongly supports the Asgard archaea clade as the closest relatives of all eukaryotes (Nikolayev et al. 2020).

One enzyme participating in this pathway is the Golgi endo-α-1,2-mannosidase. The purpose of its activity is to enable a higher proportion on N-glycans to be processed to complex structures, adding “polish” to the N-glycans. It is localized to *cis*-Golgi and the ER-Golgi intermediate compartment (Zuber et al. 2000). As the only endo-acting mannosidase in this organelle, its catalytic activity is unique. Here, an attempt will be made at answering the question why endomannosidase processing might be necessary for unicellular and multicellular organisms.

**Fig. 1:**
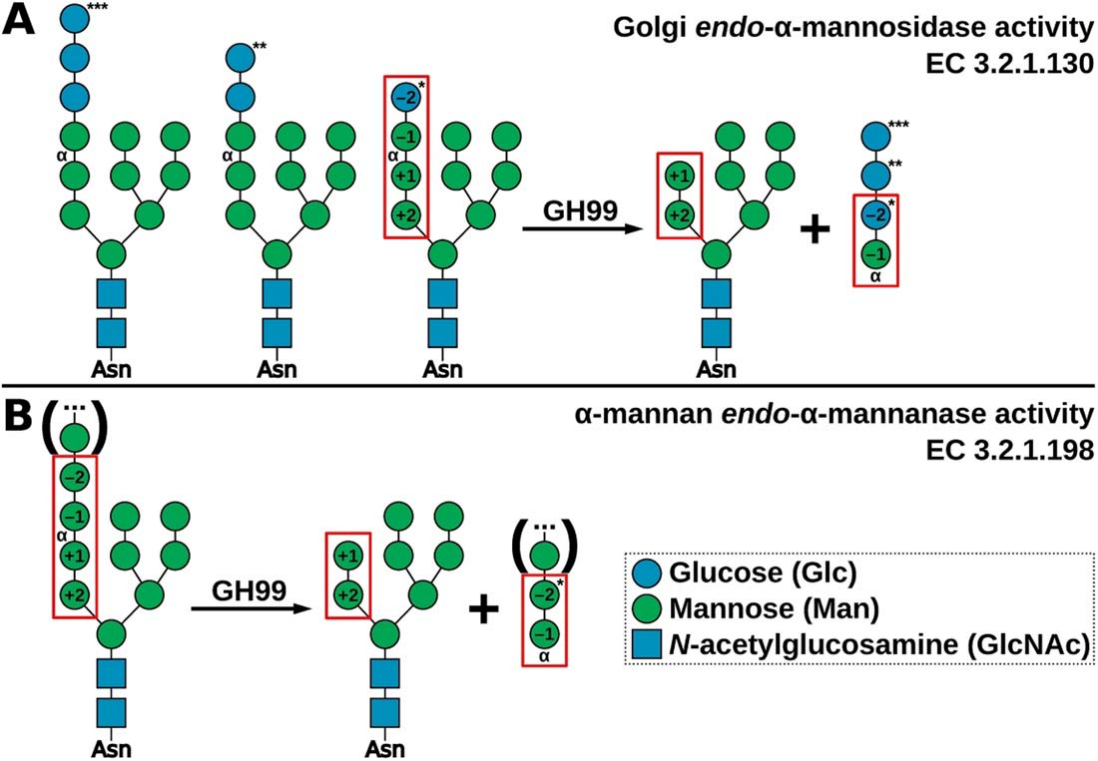
Reactions catalyzed by the GH99 endomannosidases and endomannanases. (A) N-glycan cleavage by the Golgi endomannosidase; (B) α-mannan cleavage by an endomannanase. Corresponding substrate and product variants are marked with the same number of asterisks. The minimal substrate and minimal products are in red rectangles. Subsite positions (Davies et al. 1997) are written inside the symbols. Monosaccharide symbols follow the symbol nomenclature for Glycans system (Varki et al. 2015; Neelamegham et al. 2019).

The Golgi endomannosidase is classified by the Carbohydrate Active Enzyme (CAZy) database (http://www.cazy.org/) in family GH99 (Drula et al. 2022). Its discovery (Lubas and Spiro 1987) explained previous observations of biosynthetic N-glycan trimming despite inhibition of glucosidase II (Saunier et al. 1982; Romero et al. 1985) and Golgi mannosidase I (Tabas and Kornfeld 1979; Tulsiani et al. 1982). In vivo, the animal endomannosidase catalyzes the removal of di-, tri-, or tetrasaccharide from the long arm of the precursor N-glycan attached to a glycoprotein (Fig. 1A). The preferred minimal substrate of the enzyme is a 3αGlc-2αMan-2αMan-Man tetrasaccharide, which is hydrolyzed by the endomannosidase to 3αGlc-Man and 2α-mannobiose (Fig. 1A). The preference of the Golgi endomannosidase for di- and triglucosylated substrates, as well as mannosylated substrates, is lower. Indeed, the bovine endomannosidase was found to restrict its processing to monoglucosylated substrates (Kukushkin et al. 2012). The reaction mechanism of a related protein, bacterial endomannanase from family GH99 was recently described in detail (Sobala et al. 2020b). The catalytic residues in the human MANEA (gene *MANEA* named after: MANnosidase Endo-Alpha) enzyme are E404 and E407—identical to bacterial GH99 enzymes.

GH99 domain enzymes have been classified into two groups: endo-α-1,2-mannosidases and endo-α-1,2-mannanases. The term “endo-α-1,2-mannosidase” refers to all *endo*-acting glycoside hydrolase (GH) enzymes from family GH99, while “endo-α-1,2-mannanases” are enzymes from this family that prefer substrates with mannose in the –2 subsite; see Fig. 1B (Thompson et al. 2012; Cuskin et al. 2015; Hakki et al. 2015). EC number 3.2.1.130 refers to the “glycoprotein endo-α-1,2-mannosidase” (International Union of Biochemistry and Molecular Biology Nomenclature Committee 1992). This term encompasses the enzymes present in the Golgi in animals, as well as the studied bacterial GH99 GHs from genera *Bacteroides*. These bacterial enzymes can also cleave N-glycans from glycoproteins (Thompson et al. 2012). Activity of a *Shewanella amazonensis* GH99 protein was demonstrated only on glucosylated substrates, but it is highly likely to also process mannosylated substrates (Matsuda et al. 2011). The “α-mannan endo-1,2-α-mannanase” activity is classified as EC 3.2.1.198.

Here, the term “endomannosidase” will be used to refer to enzymes that preferentially process −2-glucosyl substrates and “endomannanase” will refer to enzymes that prefer −2-mannosyl substrates. This preference is largely determined by the presence of Tyr (for endomannosidases) or Trp (for endomannanases) at position 189 (Thompson et al. 2012; Hakki et al. 2015; Sobala et al. 2020a). The *Shewanella* GH99 contains a nonpolar Leu residue at position 189—more likely to resemble Trp. In this work, an assumption is made that the predicted substrate preference (mannose or glucose [Glc] in the −2 subsite) of the endomannosidase may be used as a proxy pointing to its usage by cells. Another motif important for substrate preference is the “–2 loop,” first recognized in the human endomannosidase (residues 195–199), which was found to form hydrogen bonds with −2 subsite Glc (Sobala et al. 2020a).

According to sequence similarity, the GH99 GH family is most closely related to GH71 (Huo et al. 2017). Proteins from family GH71 are present in many *Actinobacteria* (Costa et al. 2020) and fungi and are involved in glucan degradation (Wiater et al. 2005). The similarities of these families do not end with sequences, as GH71 enzymes (mutanases) share with GH99 a tetrasaccharide minimal substrate and *endo*-activity (Grün et al. 2006). Unlike GH99, the reaction mechanism of GH71 domain proteins was not studied and the catalytic residues are unknown, but it is known that the enzyme is inverting. The second most closely related family is GH25 (Huo et al. 2017), which includes lysozymes, also present in some bacteria (Bachali et al. 2002; Ragland and Criss 2017).

Despite the importance of N-glycosylation, the evolutionary trajectories which shaped this pathway were rarely studied in detail, although notable comparative studies exist (Banerjee et al. 2007; Schiller et al. 2012; Paschinger and Wilson 2015; West et al. 2021). In particular, the evolutionary history of the endomannosidase remains obscure: so far, only two works were published. In an early taxonomic survey of biochemical activity (Dairaku and Spiro 1997), the authors concluded that the endomannosidase was a relatively recent addition to the N-glycan trimming enzyme repertoire, absent from plants, *Tetrahymena*, *Trypanosoma*, *Leishmania,* and yeast. All vertebrates were found to possess endomannosidase activity. The only invertebrates investigated in this study were arthropods and mollusks, and it was stated that nonchordates lack this activity with a “conspicuous” exception of mollusks. In a more recent, small taxonomic study (Sobala 2018) it was stated that while many insects and other invertebrates do contain the endomannosidase, sponges, and ctenophores lack this protein. It was also argued that bony vertebrates contain two separate genes encoding for GH99 domain proteins, *MANEA* and *MANEAL*, while cartilaginous fishes possess only one gene. However, the taxon sampling in those two studies was limited. The functions of GH99 domain proteins from eukaryotes less closely related to humans were never studied. Possible functional variations of various clusters of GH99 domain proteins were mentioned in a recent review (Burchill et al. 2023), but all eukaryotic proteins formed a single cluster and the evolution of their sequence motifs was not investigated further.

Relevant to the current article, the timing and character of the two genome duplications that happened early in vertebrate evolution (Dehal and Boore 2005) have been resolved recently via an analysis of deeply conserved synteny (Simakov et al. 2020). The 17 discovered linkage groups were later revised to 18 (Nakatani et al. 2021; Simakov et al. 2022) present in the ancestral chordate, but 17 were present in a protovertebrate immediately before the first whole genome duplication (1R). 1R happened in an ancestor of all extant verterbates (but not in nonvertebrate chordates: lancelets and tunicates). The second duplication (2R) took place in an ancestor of all jawed vertebrates (gnathostomes). While 1R (an autotetraploidy) led to symmetrical gene loss, 2R was a result of hybridization (allotetraploidy) of organisms α and β; the subsequent diploidization was asymmetrical. A common ancestor of a major group of vertebrates, teleost fishes, experienced a third round of whole genome duplication: 3R (Amores et al. 1998; Betancur-R et al. 2017; Pasquier et al. 2017; Chen et al. 2018; Hughes et al. 2018; Lin et al. 2020). It was a result of another autotetraploidy, but the subsequent gene loss was not symmetrical (Parey et al. 2022).

The availability of sequencing data today allows us to re-analyze and extend these findings under a greater evolutionary context and a wider taxon sampling. Here, a taxon-rich bioinformatic survey to decipher the origin and the evolutionary history of GH99 domain proteins was performed. Its results show that contrary to previous suggestions, the endomannosidase is widely distributed in eukaryotes, and in animals it is not limited to vertebrates but almost ubiquitous. Phylogenetic analyses enabled tracking the duplication history of the endomannosidase and discovering the existence of a previously unrecognized vertebrate endomannosidase paralog, present only in certain fish. In this article, the amino acid (AA) numbering of the human MANEA protein is used (UniProt ID **Q5SRI9**; identifiers shown in bold) as a reference when discussing motif evolution and protein variants from other species. Moreover, an assumption made for this analysis is that when a predicted protein with the GH99 domain is found in a particular organism, this implies the presence of a gene encoding for this protein in its genome. These genes will be referred to as “GH99 genes,” and the corresponding proteins as “GH99 proteins.” The names of GH99 genes will be italicized and the names of GH99 proteins will not be.

## Results

### GH99 proteins are widespread in eukaryotes

**Fig. 2:**
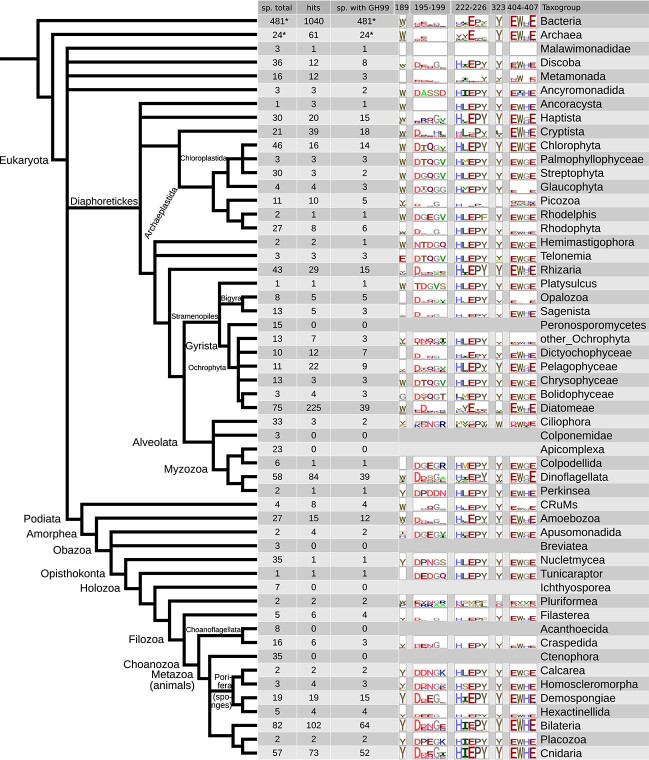
Analysis of the prevalence of GH99 proteins in the LukProt+Picozoa+*Txikispora* database and the evolution of its five main sequence motifs. Asterisks denote taxogroups (Richter et al. 2022) where the absolute prevalence could not have been estimated because of the unknown number of searched species (sp.). Sequence logos created using ggseqlogo (Wagih 2017). Only sequences not deemed as “contamination” after multiple rounds of phylogeny-based decontamination were used to build the sequence logos. In the table, the proportion of species with GH99 proteins is slightly lower than in the full database because the clustering method used (cd-hit at 95% identity) eliminated hits from some highly similar species.

The present survey found GH99 proteins in many clades of bacteria, archaea, and eukaryotes. In the searched databases (see Materials and methods), the only eukaryotic clades that did not appear to have any GH99 proteins were Colponemidae, Peronosporomycetes, and Apicomplexa, all within the broad Diaphoretickes group (Fig. 2). Due to data availability, the taxon sampling was very low for Colponemidae, which makes this conclusion tentative. In Podiata (Cavalier-Smith 2013), a clade that includes animals, fungi, and amoebas, GH99 genes are present in some form in all groups except Breviatea, Ichthyosporea, Acanthoecida, and ctenophores. The low number of sampled breviates (3) leaves open the possibility that they possess GH99 genes, but the other clades likely suffered secondary GH99 gene losses. These secondary losses are relatively common in eukaryotes: for example, no land plants have GH99 genes but two species of Streptophyta have them. One of them is *Cylindrocystis brebissonii* from a green algae clade Zygnematophyceae, which is sister to land plants.

The GH99 protein family underwent a considerable expansion and diversification in diatoms (Diatomeae), some of which have a high number of hits per species. An extreme example is *Nitzschia sp.* RCC80 (Marron et al. 2016), in which 21 predicted proteins (clustered at 95% identity) were found. GH99 protein sequences from diatoms form two well-supported major clades (Supplementary Fig. S1).

### GH99 proteins have two main predicted activities

**Fig. 3:**
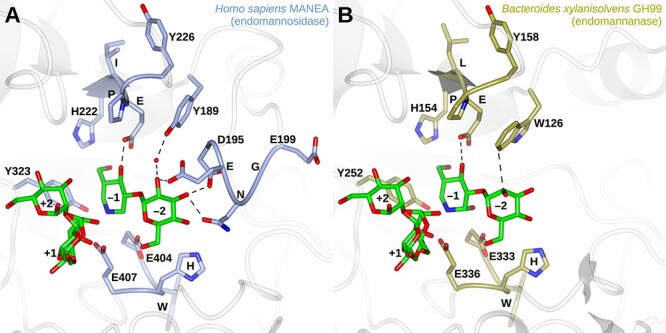
Comparison of the main motifs of the catalytic domains of (A) human GH99 (MANEA, PDB ID: **6ZFA**) and bacterial GH99 (PDB ID: **5M03**) and their substrate modes of binding. Each protein is complexed with a disaccharide analog in subsites −2/−1 and 2α-mannobiose in subsites +1/+2. All five investigated motifs can be seen in (A). Sugars are numbered according to the subsite (Davies et al. 1997) they occupy. Figure prepared in ccp4mg (McNicholas et al. 2011).

As a tryptophan was observed as residue 189 in the highest number of organisms, in most clades the endomannosidase is predicted to prefer a Man4 tetrasaccharide minimal substrate (Thompson et al. 2012) (Fig. 3B) that interacts hydrophobically with the −2 mannose (Fig. 3B). The second most common residue 189 was tyrosine, which switches the −2 sugar residue preference to Glc by forming a water-mediated hydrogen bond with the −2 sugar hydroxyl group (Sobala et al. 2020a; Burchill et al. 2023) (Fig. 3A).

Among bacterial GH99 proteins, the most common residues at position 189 were: W (63%), Y (19%), and L (10%). In Archaea an even higher proportion contained W189—the values are, respectively: 85%, 3%, 2% (Supplementary Table S6). It is known that Bacteroidetes use the endomannanase to digest yeast mannan (Cuskin et al. 2015). The emergence of the GH99 domain, which implies existence of the endomannanase activity, happened earlier than the divergence of fungi over 1 billion years ago (Strassert and Monaghan 2022). This is evidenced by the observed ubiquity of GH99 genes. Thus, endomannosidase/endomannanase activity evolved much earlier and cellular organisms probably first utilized this activity to remove recalcitrant branches of various exogenous mannose-containing glycans. This would make the smaller oligosaccharides available for downstream exoglycosidases, which would then release monosaccharides ready for use in the cell’s own metabolism. It is likely that the first eukaryotes used the catalytic activity of GH99 proteins in a similar way to extant bacteria. This is also evidenced by finding of a number of partial, reconstructed GH99 proteins in Ancyromonadida, which are reported as an early diverging lineage of eukaryotes (Brown et al. 2018). These proteins contain all the crucial active site residues, as well as W189, signifying the ancient origin of this catalytic activity.

The next motif, spanning residues 195–199 (**D**[E/D]N**G**E, part of the “–2 loop”; highly conserved residues shown in bold) seems to be a common feature in eukaryotic GH99 proteins, with D196 and G198 conserved in many clades. The notable exceptions are Haptista where only G198 is conserved, Cryptista with a partial consensus motif, and diatoms with only D196 conserved. The third motif H[I/L]**E**PY (residues 222–226) is conserved in all clades except Diatomeae and Metamonada. The fourth motif, Y323, is indeed quite conserved but does not seem necessary for catalysis. For instance, it was substituted for F323 in full length MANEAL proteins in certain fish species. Finally, the fifth motif (404–407) containing two catalytic site glutamic acid residues, **E**W[H/G]**E**, is conserved across all domains of life (except Picozoa, for which the source sequences might be less accurate).

Building upon the understanding of the structure and motif evolution in the GH99 domain, it was compared to its closest CAZy family, GH71, which is also present in bacteria and eukaryotes. The high overall prevalence of GH71 and GH99 genes suggests they diverged before Archaea separated from bacteria. A manually constructed alignment of human GH99 protein (MANEA) and an AlphaFold GH71 protein structure (Jumper et al. 2021) of *Emericella nidulans* mutanase (UniProt ID **Q96VT3**) is included (Supplementary Fig. S5). The GH71 protein structure is similar, with a (β/α)_8_ barrel as the core. The alignment reveals chemically similar candidate active site residues and commonalities in ligand binding between the two GH families, with a distinct **D**YG**E** consensus active site motif instead of the **E**WH**E** of GH99. *E. nidulans* GH71 does not have a residue analogous to Y189 of GH99 proteins, but a conserved and spatially close N74 may play a similar role in substrate binding. Interestingly, some GH99 proteins also contain the **D**YG**E** motif, for example a sequence from a holozoan *Syssomonas multiformis* and multiple proteins from a dinoflagellate species *Kryptoperidinium foliaceum*.

### Only select lineages use GH99 proteins in their Golgi N-glycan processing pathways

**Fig. 4:**
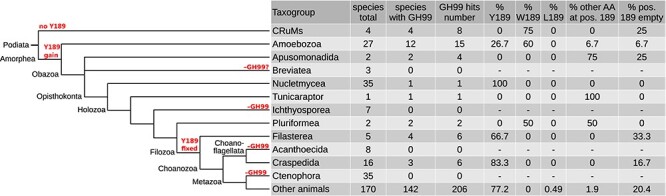
A W189 to Y189 switch observed within the Podiata clade. Major changes in the GH99 protein repertoire or Y189 status are marked in red. Percentages refer to all GH99 proteins found in each clade.

In Amorphea, within which animals are contained and whose sister group is CRuMs (Brown et al. 2018) (Figs. 2 and 4), a switch from W189 to Y189 was observed. In none of the GH99 proteins from CRuMs (4 taxa sampled) Y189 was present, but a significant proportion (4 sequences, 26.7%) of hits from Amoebozoa have this feature (Fig. 4, Supplementary Table S6). Some amoebozoans possess GH99 genes encoding for both W189 and Y189 GH99 proteins, suggesting the latter arose from a gene duplication in their ancestor. In Obazoa, a sister clade to Amoebozoa, only Opisthokonta retained Y189 and among the opisthokonts, the only nucletmycean that contains it is the recently sequenced *Parvularia atlantis* (Ocaña-Pallarès et al. 2022). No true fungi possess GH99 proteins. Among the closest relatives of animals, the facultatively multicellular holozoans, Tunicaraptor and Pluriformea have various residues at site 189. Filozoa is the first clade where Y189 is prevailing (66.7%) with no other residues found at this position, not counting partial sequences where this residue is missing (Figs. 2 and 4). In craspedid choanoflagellates this Y189 is found in 83% of all sequences, and in non-ctenophore animals in 77%. Thus, it appears that Y189 is a feature of the last common ancestor of Filozoa. For a targeted analysis of the *Tunicaraptor unikontum* GH99 protein see the associated Zenodo repository.

The W189 → Y189 switch also happened in Rhizaria (Fig. 2) in the Chlorarachniophyta group. For example, the model rhizarian *Bigelowiella natans* as well as *Chlorarachnion reptans* have two GH99 sequences, one with W189 and one with Y189. These sequences have different 195–199 loop structures, further pointing to differences in their substrate specificity. In these species, W189 is coupled with an **E**WG**E** active site motif and Y189 with **E**WH**E**. The W189/**E**WG**E** sequences are most similar to GH99 sequences from Cryptista and Chlorophyta, suggesting they are descendants of the sequence from the phototrophic endosymbiont (Oborník 2019) (Supplementary Fig. S1). The Y189/**E**WH**E** sequences come from the rhizarian host. Another group where the switch also happened may be Picozoa, although sequences from them might be less reliable as sequences from these species come mostly from single-cell genomes.

### All nonbilaterian animal phyla except Ctenophora possess GH99 genes

Analysis of the extended LukProt dataset (see Materials and methods) showed that all the major animal clades, except Ctenophora (Fig. 4), possess GH99 genes. The sequences found in sponges, placozoans, and cnidarians are always *MANEA*; indeed, no evidence was found of GH99 gene duplications, or existence of *MANEAL*, in any major clades of non-gnathostomes. Sponges, placozoans, and cnidarians usually possess one copy of the gene per species.

Strikingly, and contrary to previous claims (Dairaku and Spiro 1997; Sobala 2018), endomannosidases were found in all four classes of sponges. In phylogeny reconstructions GH99 proteins from sponges reproduce the topology of canonical sponge phylogeny (Wörheide et al. 2012). In a number of demosponges and homoscleromorphs more than one GH99 protein copy per species was found. In *Stylissa carteri* and *Halichondria panicea* they are products of the same gene. However, in demosponges *Cymbastela concentrica* and *Spongia officinalis*, analysis of a multiple sequence alignment (see Data Availability Statement) suggests they are products of different genes. Crucially, they possess all five canonical motifs presented in Fig. 2. In summary, despite losses in individual lineages, GH99 proteins with predicted endomannosidase activity seem to be a fixture among sponges, placozoans, and cnidarians (except the highly specialized, parasitic myxozoans, see Supplementary Table S1), but ctenophores do not possess it.

One potential and ancient instance of lateral gene transfer was detected: from an ancestral Cnidarian/Placozoan to a common ancestor of two algae from the group Chattonellales—*Heterosigma akashiwo* and *Chattonella subsalsa* (Supplementary Fig. S1). Sequences from these species form a well-supported clade and the algal GH99 proteins contain the conserved animal residue Y189, while other sequences from ochrophytes mostly do not. This gene transfer could have occurred between a hypothetical ancestor of both Placozoa and Cnidaria (Simakov et al. 2022) and a common ancestor of Chattonellales. Further studies and timed phylogenies of Raphidophyceae are needed to investigate this possibility.

### All major clades of bilaterians except the sampled Xenacoelomorpha possess GH99 genes

**Fig. 5:**
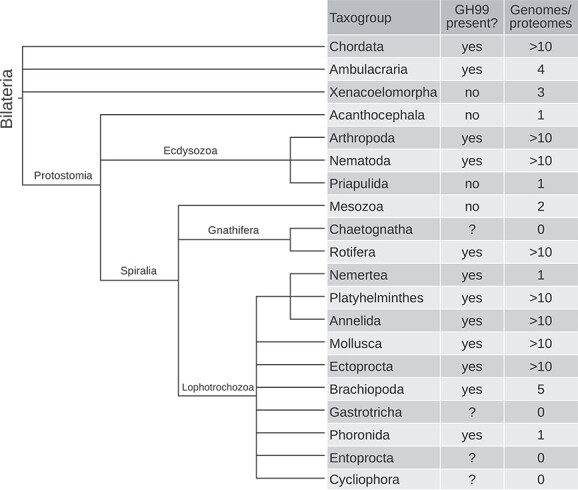
Cladogram and presence/absence analysis of the GH99 proteins in bilaterian taxa, with emphasis on nonchordates. Clades are based on (Marlétaz et al. 2019) and information from NCBI taxonomy (Schoch et al. 2020).

A survey of bilaterian clades yielded similar results: GH99 genes can be found in almost all. The average fraction of species that contained one or more clustered GH99 proteins from LukProt was: 70–100% in arthropods, lophotrochozoans, ambulacrarians, and nonvertebrate chordates, lower in nematodes (40%) and 0 in xenacoelomorphs. The number of Xenacoelomorpha taxa sampled was three, too low to exclude the possibility of the gene existing in other xenacoelomorphs (Fig. 5).

By extending the search to all sequences available in the NCBI “nr” database, as well as TBLASTN searches on genomes for which predicted proteins are not available (Supplementary Table S1) a diverse sampling of protostomes was surveyed. The single acanthocephalan genome did not contain any GH99 genes. Rotifers do possess GH99 genes but the status of their probable sister clade Chaetognatha (Marlétaz et al. 2019) is unknown, as there are no genomes available (Fig. 5). Among lophotrochozoans, all clades with a genome available have GH99 genes. The ecdysozoan clades that contain GH99 genes are Nematoda and Arthropoda. Sequences from Diptera (arthropods—flies) formed two distinct clades in the complete phylogeny (Supplementary Fig. S1). Both urochordates and hemichordates (Ambulacraria) possess the GH99 genes, as well as all the surveyed chordate clades. In summary, GH99 genes are widely distributed in bilaterians and only select clades do not contain any species with it.

### *MANEA* and *MANEAL* originated in the second round of vertebrate whole genome duplication

The taxonomic survey of GH99 protein phylogenies (Supplementary Figs. S2 and S3, coloring scheme: Supplementary Fig. S7) largely agrees with the auto-then-allotetraploidy model of vertebrate evolution. Non-gnathostomes possess one GH99 gene, whereas most gnathostomes possess two genes, *MANEA* and *MANEAL*.

**Fig. 6:**
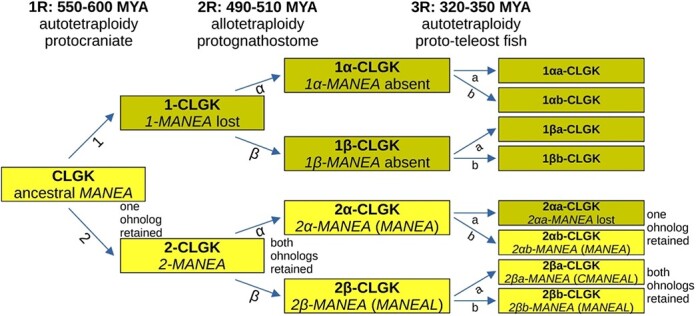
Duplication history of vertebrate GH99 genes in context of the known whole genome duplications. The duplication dates, CLG and duplication terminology is taken from (Jaillon et al. 2004; Simakov et al. 2020; Parey et al. 2022). Duplicated genes and CLGs are prefixed to help trace their origin. Accepted and proposed gene names are written in brackets. Bright yellow blocks symbolize CLGs which contain a GH99 gene and dark yellow blocks are CLGs that do not contain GH99 genes.

A closer examination of the data published with the vertebrate synteny analysis (Simakov et al. 2020) reveals that *MANEA* was located on the ancestral protovertebrate chromosome Conserved Linkage Group K (CLGK). *Branchiostoma floridae* chromosome BFL9 is the direct descendant of CLGK and in this amphioxus *MANEA* is localized there (Fig. 6). The chromosomal locations of *MANEA* and *MANEAL* in other surveyed species (human, chicken, western clawed frog) help triangulate the origins of *MANEA* and *MANEAL* ohnologs (paralogs resulting from whole genome duplications): *MANEA* comes from organism α and *MANEAL* from organism β (Simakov et al. 2020) (Supplementary Table S2).

Members of the clade of cartilaginous fishes, which diverged from other jawed vertebrates early, all have both *MANEA* and *MANEAL*. Among ray-finned fishes, non-teleosts have the two GH99 genes, but teleosts from the Clupeocephala supercohort usually have three (Fig. 6). This third vertebrate GH99 protein they encode for consistently forms a separate clade in phylogenies, sister to the MANEAL clade (Supplementary Fig. S2). It is always absent in their sister teleost clade, Eloposteoglossocephala (Parey et al. 2023). In addition, it is found on the “2a” homeolog (duplicated chromosome) in clupeocephalans, while *MANEAL* is found on “2b” (Parey et al. 2022). This strongly suggests that it is a paralog of *MANEAL* that arose from 3R and was retained clupeocephalan fishes but lost in Eloposteoglossocephala. Taking this into account, the clupeocephalan paralog of *MANEAL* is tentatively named Clupeocephala *MANEAL* (*CMANEAL*), while *MANEAL* is retained as the name of the gene with more ancestral features. No further GH99 gene duplications retained in major vertebrate clades were detected.

The phylogenetic analysis mentioned above unambiguously places GH99 proteins in the MANEA, MANEAL, or CMANEAL clades. As automatic gene annotation is especially prone to errors resulting from gene paralogy, many genes that encode for MANEA have been mislabeled as MANEAL. Supplementary Table S3 contains a list of misclassified vertebrate GH99 proteins and Supplementary Table S4 contains a list of all classifications of vertebrate GH99 proteins. Supplementary Table S7 contains lists of vertebrate species with only *MANEA*, only *MANEAL*/*CMANEAL,* or with both.

### Among vertebrates *MANEA* is rarely lost but *MANEAL* loss is common

Analysis of gene presence in vertebrates showed that *MANEA* is hardly ever lost in this group. Uniquely, the only vertebrates that lost *MANEA* are the bowfin *Amia calva* and the albatross *Thalassarche chlororhynchos* in whose genomes this gene could not be detected. The catalytic activity of an endomannosidase seems to be essential for vertebrate survival, as both of these species possess GH99 genes encoding for *MANEAL* proteins which are predicted to be active.

**Fig. 7:**
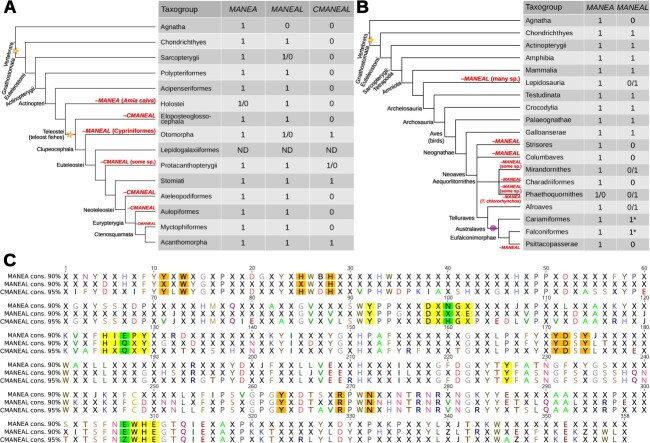
Analysis of vertebrate *MANEA*, *MANEAL,* and *CMANEAL*: gene presence/absence and consensus (cons.) protein sequences. (A) Actinopterygii-centric cladogram, (B) Sarcopterygii-centric cladogram, (C) Alignment of catalytic domain consensus sequences (at 90% or 95% identity). Asterisks denote probable instances of pseudogenization, stars signify gene duplication events and the spiral is a pseudogenization event. The only two instances of *MANEA* loss in vertebrates are marked in red. In (C) the five investigated motifs are highlighted in yellow and crucial differences between them in the three orthologs are highlighted in green. Other residues in close proximity to the substrate are highlighted in orange. Catalytic domain residue numbering is shifted by −97 with respect to human MANEA. For full phylogenies refer to Supplementary Figs. S2 and S3. Simplified fish phylogeny based on (Betancur-R et al. 2017) and bird phylogeny based on (Prum et al. 2015; Sangster et al. 2022).

In contrast, *MANEAL* seems to be more dispensable. In clupeocephalan fish, whose last common ancestor had *CMANEAL*, large clades acquired predicted inactivating mutations or gene losses. Cypriniformes, an order of Otomorpha, lost *MANEAL* but retained *CMANEAL* (Fig. 7A, Supplementary Fig. S2). In a major clade of clupeocephalans, Acanthomorpha, CMANEAL proteins suffered a probable loss of function mutation similar to artificially created GH99 inactive variants described earlier (Thompson et al. 2012; Iwamoto et al. 2017; Sobala et al. 2020b) (E404Q). Their MANEAL proteins were consistently the active variants (E404), suggesting that MANEAL is catalytically active in these species but CMANEAL may not be. TBLASTN searches of the three available genome assemblies of Acanthomorpha cousins within the clade Neoteleostei suggested that only vestiges of *CMANEAL* are present in their genomes but they all seem to retain *MANEA* and *MANEAL* (Fig. 7A).

As the branches in the CMANEAL subtree are not long (Supplementary Fig. S2), it is possible that the protein gained a different function in species possessing the *CMANEAL* gene. Short branches suggest some selective pressure against random mutations, which would have not been seen if the protein was dispensable and pseudogenized.

Another consistent feature of CMANEAL is that the consensus sequence of the −2 motif is D[D/E]**H**GE instead of D[D/E]**N**GE present in other animal GH99 proteins (Fig. 7C). As residue N197 forms a hydrogen bond with the O4 of the −2 Glc (Sobala et al. 2020a) (Fig. 3), the N197H variation might change the substrate binding affinity, possibly increasing it. This is predicted because the electronegativity difference would be greater in case of the NH–O bond than in case of the OH–O bond. It is also possible that in Clupeocephala there exists some evolutionary pressure to develop stronger binding to the −2 sugar. Interestingly, in two clades of fish (Siluriformes and Characiformes), it is MANEAL which is the N197H variant and CMANEAL is an E404 variant, predicted to be active. In their sister group, Cypriniformes, *MANEAL* was lost (Fig. 7A, Supplementary Fig. S2). Therefore, E404 CMANEAL and MANEAL might have redundant activities, and genes for either can be lost or evolve different functions.

The loss of *MANEAL* also happened multiple times in various lineages of tetrapods. In Amphibia (amphibians) all major orders possess both genes. However, in squamates, the largest order of lizards, *MANEAL* appears to have been independently lost in geckos (Gekkota), Pleurodonta, and snakes (with a caveat that there are no genomic data available for thread snakes, blind snakes, and amerophidians) (Supplementary Fig. S3). The overall availability of sequencing data in lizards is low and these results are tentative. The only living non-squamate reptile, the tuatara, possesses both *MANEA* and *MANEAL*.

Among birds, *MANEAL* losses are common but their pattern is more scattered. The major bird clades lacking *MANEAL* are nightbirds (Strisores) and Columbaves (Fig. 7B). All fowl (Galloanserae) contain both proteins. Among Neoaves, within the large waterbird clade Aequorlitornithes, both proteins are retained in some species in the subclades Mirandornithes and Phaethoquornithes, but the major clade Charadriiformes lacks *MANEAL* (Fig. 7B). In Telluraves (a Neoaves subclade), *MANEAL* was lost in Psittacopasserae (passerines + parrots), but in their closest cousins, falcons (Falconiformes) and seriemas (Cariamiformes) MANEAL protein sequences with mutations in the active site were detected. For example, falcon MANEAL predicted proteins are N-terminally truncated and have a mutated active site (**E**WH**E**→**K**WH**E**). *MANEAL* therefore probably pseudogenized is the last common ancestor of Australaves and was subsequently lost from the genome in the last common ancestor of Psittacopasserae (Fig. 7B). Among Afroaves many species lost *MANEAL*, but some (spotted honeyguide, keel-billed toucan) retained it.

Tetrapod phylogenies also uncovered that in the bird and lizard species which retained *MANEAL*, the gene underwent faster change than *MANEA*. While the MANEA predicted protein branch is largely representative of true phylogenetic relationships between species (Supplementary Fig. S3), the MANEAL clade is not: squamate MANEAL proteins were incorrectly placed inside the Archelosauria clade with a high UFBoot2 (ultrafast bootstrap 2) support of 97. The crocodile MANEAL clade was artefactually placed inside the bird MANEAL clade (UFBoot2: 62) and the branches were long. The probable falcon pseudogenic MANEAL proteins formed a tight cluster on a particularly long branch. Many other archelosaurian MANEAL protein sequences are found on long branches, supporting the pseudogenization hypothesis (Supplementary Fig. S3).

Analysis of the CMANEAL clade (Supplementary Fig. S2) suggests that despite suffering a probable inactivating E404Q mutation, in most species the gene likely did not pseudogenize. Such a process would have been visible as an abundance of long branches resulting from sequences that acquired random mutations. In Fig. 7C an alignment of the consensus catalytic domains of animal vertebrate MANEA, MANEAL, and CMANEAL is presented. In addition, for each of the three ohnologs, a separate catalytic domain profile was prepared. The protein hits were found using HMMER and the difference in e-value was plotted for each hit that all models detected. This resulted in easily identifiable clusters representing MANEA (together with ancestral sequences), MANEAL, and CMANEAL (Supplementary Fig. S4).

Further proteome analysis results show that all mammals have both *MANEA* and *MANEAL* genes. In the cases where both of the proteins were labeled with the same name (MANEAL), through phylogenetic analysis these were all found to be annotation errors. A number of mammalian genome annotations (12 species) suggested they only possess *MANEA* and 6 other annotations state *MANEAL* only. TBLASTN searches of each of these assemblies (Supplementary Table S1) and BLASTP searches of the translated protein models were conducted to test this. In every case both *MANEA* and *MANEAL* genes were found.

## Discussion

### Evolution of the GH99 domain

In this work the evolution of motifs in GH99 proteins and abundance of GH99 genes in various taxonomic groups placed in their phylogenetic context is described. The conclusions of both of the initial attempts at the analysis of endomannosidase origins (Dairaku and Spiro 1997; Sobala 2018) were tested and found incomplete and in some cases incorrect. The authors of the early taxonomic survey did not enjoy the current abundance of sequencing data and sampled only 20 taxa; in the current analysis, hundreds of species were included. The data presented here suggest that the endomannosidase activity is present in many lineages of nonvertebrates, as well as almost all clades of eukaryotes. This conclusion is drawn only by inference from sequence similarity. GH99 proteins in some lineages might not be endomannosidases or endomannanases, but they are a minority—care was taken to look at key motifs that determine the enzymatic activity. Ultimately these findings can be tested by biochemical studies comparing sequences with different active site motifs. The specific claims (Dairaku and Spiro 1997) refuted by this analysis are: (i) that endomannosidase is a recent addition to the eukaryotic N-glycan processing machinery, (ii) that it is limited in distribution to chordates with the exception of mollusks. Contrary to the later research (Sobala 2018), it is also evident that GH99 genes *MANEA* and *MANEAL* are present in all clades jawed vertebrates—not only in bony vertebrates.

As bacteria, archaea and most clades of eukaryotes possess GH99 genes, the last eukaryotic common ancestor probably also possessed a GH99 gene. Motif conservation (Fig. 2) suggests that the form of the enzyme containing all the five sequence motifs typical to animal GH99 proteins emerged early, probably in a common ancestor of all eukaryotes. It would be a remarkable case of convergent evolution if they appeared separately in most Diaphoretickes and Podiata. The preference toward GluMan_3_ (Y189) became fixed in the ancestral filozoan and is conserved in most species from this clade. The last common ancestor of all animals posessed one copy of the ancestral *MANEA* gene and all major animal clades, except the ctenophores, inherited it.

### From endomannanase to endomannosidase

The present data allows speculation as to why the Y189 form (endomannosidase) is conserved in almost all animals, while throughout eukaryotes the prevailing form is W189 (endomannanase). The precursor glycan, while shorter in some species, has the Glc_3_Man_9_GlcNAc_2_ structure in groups as distant as plants and animals (Banerjee et al. 2007; Schiller et al. 2012). Shortening of the precursor is predicted to be an effect of secondary losses of glycosyltransferases within eukaryotes (Schiller et al. 2012). Why did most eukaryotes not optimize their GH99 proteins toward the glucosylated N-glycan? An attractive proposition is that within filozoans (see Fig. 2), the increasing complexity of life histories associated with predatory lifestyles (Hehenberger et al. 2017; Tikhonenkov et al. 2020a, b) required higher quality control mechanisms. These mechanisms potentiated filozoans and choanoflagellates to evolve clonal lifestyles and multicellularity. In multicellular organisms, cells must communicate their identity to others precisely, otherwise they could be recognized as nonself and expelled or killed. N-glycans on membrane-anchored proteins may form such signaling tags. The endomannosidase ensures that Glc is trimmed from any glycoproteins exported from the ER to the Golgi. Absence of this enzyme would cause leakage of glucosylated N-glycoproteins to the cell surface and the extracellular matrix, as well as preventing the processing of the affected glycans by any downstream glycosyltransferases. This would result in a significant reduction in the proportion of glycoproteins with hybrid and complex N-glycans.

In this view, the evolution of multicellularity depended on and potentiated the selection of well-defined (identity-signaling) cell surfaces, which in turn depended on effective cellular mechanisms of quality control. While such mechanisms are expensive for single cells in terms of energy, they have a potential to confer selective advantages to assemblies of cells (organisms) that possesses these traits. This might be why organisms with complex, multicellular lifestyles may need precision in their N-glycan processing, and this is what the endomannosidase activity provides. The Y189 form of the enzyme preferentially binds glucosylated N-glycans, the only forms that exists in the Golgi. Presumably the W189 form would not be as efficient in cleaving the Golgi N-glycans. The context for this model is that glycans are known to participate in self–nonself recognition (Varki 2017).

The case of ctenophores lacking any GH99 genes speaks to their uniqueness in the animal kingdom. It is likely that they evolved a different mechanism of dealing with precise glycan editing. The absence of GH99 in plants and fungi, which are multicellular, also seemingly contradicts the presented hypothesis. However, their immediate ancestors were not predatory, unlike the ancestor of animals. GH99 genes are also present only in three out of 16 surveyed craspedid choanoflagellates, notably in the facultatively multicellular *Salpingoeca rosetta*. This could be an artifact of low expression—for many species, only the transcriptome was available. Finally, the nucletmycean *P. atlantis* contains a GH99 protein with Y189, not W189. Therefore, the Y → W switch might have occurred as early as in the common ancestor of all opisthokonts, but this is a single species and this change might have been lineage-specific.

### Importance of vertebrate GH99 gene ohnologs

The origins of three main GH99 gene ohnologs are presented. Previously, due to automatic annotation, the identity of GH99 genes from each species was obscured in public databases. The analyses presented here should result in corrections to databases and a recognition of a new family member of vertebrate GH99 gene sequences—*CMANEAL*, specific to clupeocephalan fishes. Analysis of previous evidence (Chen et al. 2018; Lin et al. 2020; Parey et al. 2022) clearly points to *CMANEAL* being an ohnolog lost in the Eloposteoglossocephala clade, rather than independently gained by Clupeocephala. The two almost universal substitutions that characterize CMANEAL proteins, **E**404**Q** and **N**197**H**, are predicted to cause loss of its hydrolase activity and an increase in its glycan binding affinity. This suggests that the protein evolved a separate function, possibly becoming a lectin, a prediction which can be tested in biochemical studies.

As not one vertebrate species without a GH99 gene was found, the endomannosidase activity seems to be essential for survival for every vertebrate. Patterns of GH99 gene gains and losses point to the endomannosidase being an enzyme without that most animals cannot survive. When duplicated, however, GH99 genes are dispensable: *MANEAL* and *CMANEAL* are often lost or acquire inactivating mutations. As the main motifs and overall 3D structure of MANEA and MANEAL are not predicted to differ significantly, they probably play essentially the same role but in different tissues. Some evidence for tissue specificity can be found in human protein expression database GTEx (GTEx Consortium et al. 2017), where *MANEA* is expressed throughout the body and *MANEAL* mainly in the nervous system (data retrieved from https://gtexportal.org/ on 2023 March 20). Tissue bias in paralog expression was found in previous studies (Kryuchkova-Mostacci and Robinson-Rechavi 2016; Xu et al. 2019).

Three of the five prepared HMM profiles (see the Zenodo repository) can be used to readily distinguish between *MANEA*, *MANEAL,* and *CMANEAL* (Supplementary Fig. S4A) and their protein products. These profiles can be adapted by protein family databases, such as InterPro (Paysan-Lafosse et al. 2023) to find and correctly annotate these genes. In addition, the provided sequence identifier lists (Supplementary Tables S3 and S4) can be used by curators to correct the annotations directly. However, to prevent such situations in the future, a more integrated approach of gene annotation is needed, in which paralogy, evolution, and synteny are taken into account.

### The GH99 domain in context

A large expansion of GH99 proteins in diatoms (Supplementary Fig. S1) is uncovered. The only diatom whose N-glycome was studied, *Phaeodactylum tricornutum* (Baïet et al. 2011; Xie et al. 2021), happens to have only one GH99 protein (clustering within Diatomeae GH99 major clade 2) which was omitted from the bioinformatic analyses in these glycomic studies. Such an expansion, and noncanonical motifs 195–199 and 222–226 (see Fig. 2 and the associated Zenodo repository), imply functional diversification of diatom GH99 proteins.

The understanding of GH99 domain structure and evolution allows us to propose putative ligand binding residues and active site residues of a protein from a family related to GH99, a fungal GH71 mutanase. The active site residues of GH71 family proteins were unknown but seem to be the same as in some bona fide GH99 proteins. Distant but detectable evolutionary relationships such as these can be used to study GHs and generate hypotheses about their mechanisms of action.

A limitation of the current study is that pseudogenization is investigated only indirectly (through phylogeny analysis and sequence comparison). Due to the presence of transcriptomes in the LukProt database, pseudogenes might be visible by being expressed at a low level and then assembled and transcribed in silico into partial proteins. This would increase the apparent number of vertebrates having both *MANEA* and *MANEAL*. Another limitation is that in cases where TBLASTN searches of genome assemblies were run to confirm protein BLAST results, any indication of *MANEA* or *MANEAL* in the genome was counted as their presence. This could have inflated the number of species and clades in which the sequences were detected. Such an approach was selected in order to understand broad patterns of endomannosidase evolution and not its evolution in individual taxa. The functions of *CMANEAL* were also not directly investigated. Another potential weakness of the work is the high number of sequences used to produce single-gene phylogenies, which in some cases led to overparametrization. The affected single gene phylogenies were treated as guides only, with clades concordant with currently accepted species phylogeny being treated as more informative than those which grouped together unrelated species.

In summary, these findings allowed to track the evolutionary story of GH99 protein usage by various groups of organisms and speculate on the importance of endomannosidase activity in animal multicellularity. The purpose of this study was to clear the picture of GH99 protein evolution and provide the reader with an understanding of the functional importance of the endomannosidase and endomannanase activities. Finally, the author believes that misconceptions stemming from animal-centric and model organism-centric approaches are difficult to dispel and lead to erroneous assumptions which may be reproduced in literature for decades. The reader is therefore encouraged to always consider the full tree of life when looking for origins of specific cellular activities (del Campo et al. 2014).

## Materials and methods

The LukProt database version 1.4.1 (Sobala unpublished data) was used in all local BLAST+ (Camacho et al. 2009) searches (BLASTP, TBLASTN). Remote BLASTP searches were done using NCBI BLAST online service (NCBI Resource Coordinators 2017) against the nr database and remote TBLASTN searches were performed using “BLAST Genomes” option. Bacterial, archaeal and metazoan GH99 sequences from the nr database were retrieved using BLASTP against the nr database using UniProt ID **Q5SRI9** (human MANEA) with max_target_seqs 5000 and an e-value of 1 × 10^−4^. Additionally, picozoan GH99 sequences were found using EukProt v3 (Richter et al. 2022) BLAST server (https://evocellbio.com/SAGdb/EukProt/) by running BLASTP of Q5SRI9 as query against all Picozoa. Unpublished sequences from a filasterean *Txikispora philomaios* (Urrutia et al. 2022) were searched using the same method. The user interface for local BLAST+ (v2.13.0) was SequenceServer (Priyam et al. 2019). The software used for sequence alignment was MAFFT v7.508 (Katoh and Standley 2013) (L-INS-I mode), MUSCLE v3.8.425 (Edgar 2004) and v5.1 (Edgar unpublished data) or Famsa v2.2.2 (Deorowicz et al. 2016). Alignments were then trimmed using trimAl (Capella-Gutierrez et al. 2009) (gap threshold 0.05–0.1) and, in cases explained in the associated Zenodo dataset (doi:10.5281/zenodo.7470560), manually in Geneious Prime v.2022.2.2 (https://www.geneious.com). To search LukProt with a GH99 domain hidden markov model (Pfam accession **PF16317.7**) and with GH99 profiles generated in this work, HMMER v3.3.2 (http://hmmer.org) software was used. Phylogenies were prepared using IQ-TREE 2.2.0-beta (Kalyaanamoorthy et al. 2017; Hoang et al. 2018; Minh et al. 2020) and visualized in Figtree v1.4.4 (http://tree.bio.ed.ac.uk/software/figtree/) or Dendroscope v3.8.4 (Huson and Scornavacca 2012). Unless otherwise noted, the default options changed in IQ-TREE were: --msub nuclear -alrt 5000 -bb 5000 -abayes -nm 10000. Sequences were clustered at various identities (50–100%) using CD-HIT (Li and Godzik 2006; Fu et al. 2012) (command example for clustering at 95% identity: cd-hit -s 0.5 -g 1 -d 0 -T 6 -M 16000 -c 0.95). Signal peptide searches were carried out using with SignalP 6.0 (Teufel et al. 2022). In some cases, very divergent GH99 proteins were found, which may or may not be artifacts of in silico transcript reconstruction and translation. Sequences that were obviously erroneously translated were excluded from single gene phylogenies. The list of excluded sequences with the reasons can be found in Supplementary Table S5. Other sequence handling tasks were accomplished using SeqKit (Shen et al. 2016), GNU coreutils and Geneious Prime. For details of individual alignments and phylogeny reconstructions the reader is directed to the README files in the aforementioned Zenodo dataset.

## Author Contributions

Łukasz Sobala (Conceptualization [Lead], Data curation [Lead], Funding acquisition [Lead], Investigation [Lead], Methodology [Lead], Project administration [Lead], Software [Lead], Validation [Lead], Visualization [Lead], Writing—original draft [Lead], Writing—review & editing [Lead])

## Supplementary Material

Table_SI_cwad041

Table_SII_cwad041

Table_SIII_cwad041

Table_SIV_cwad041

Table_SV_cwad041

Table_SVI_cwad041

Table_SVII_cwad041

SuppInfo_rev2_cwad041

## Data Availability

Source data, scripts, and supporting data can be found in the associated Zenodo repository: https://doi.org/10.5281/zenodo.7470560.
