# Supplementary material for: Evolution and phylogenetic distribution of endo-α-mannosidase
Source: Glycobiology. 2023 May 18;33(9):687–99. doi: 10.1093/glycob/cwad041 (PMC11025385; doi:10.1093/glycob/cwad041)
Supplement: SuppInfo_rev2_cwad041 [file suppinfo_rev2_cwad041.pdf]

Supplementary Information

Supplementary Figures with Descriptions

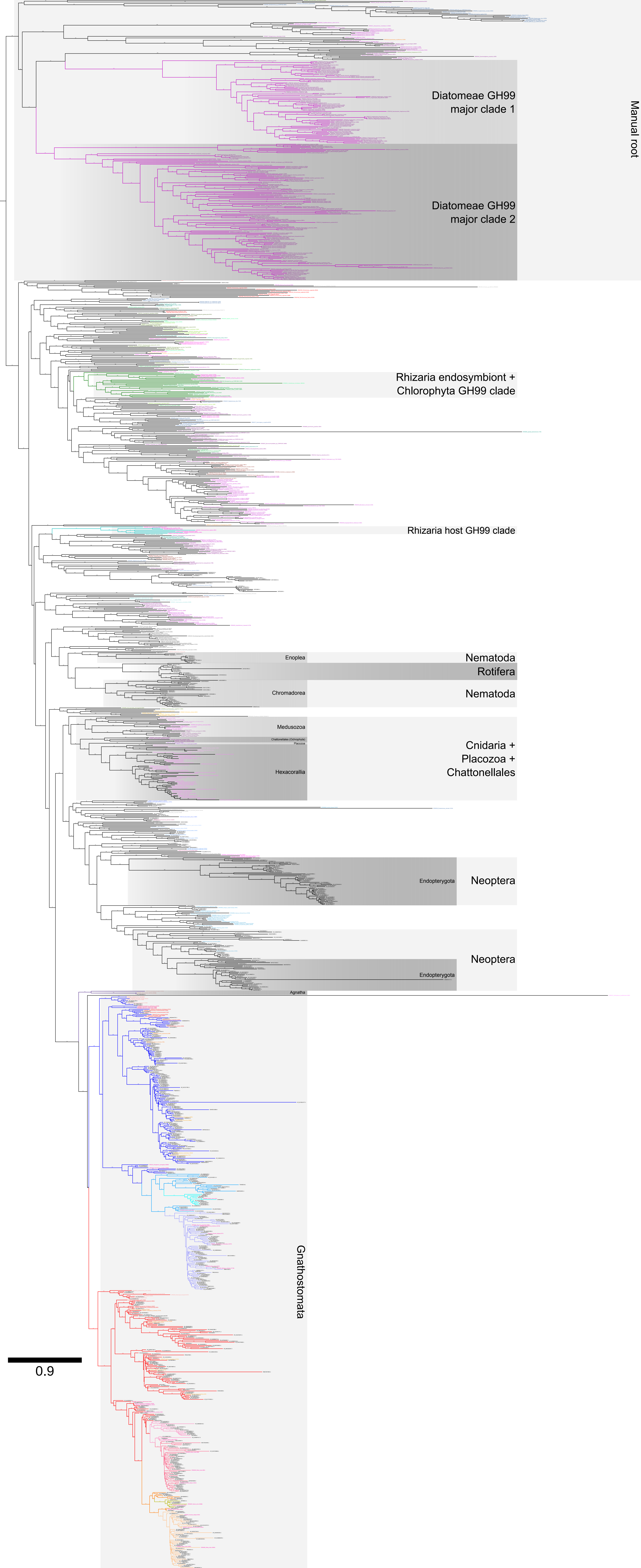

Manual root
Diatomeae GH99 major clade 1
Diatomeae GH99 major clade 2
Rhizaria endosymbiont + Chlorophyta GH99 clade
Rhizaria host GH99 clade
Enoplea
Nematoda
Rotifera
Chromadorea
Nematoda
Medusozoa
Chattonellales (Ochrophyta)
Placozoa
Hexacorallia
Cnidaria + Placozoa + Chattonellales
Endopterygota
Neoptera
Neoptera
Endopterygota
Agnatha
Gnathostomata
0.9

Supplementary Fig. S1: Annotated phylogeny all eukaryotic GH99 sequences, decontaminated, clustered at 95% identity, prepared using IQ-TREE 2. Tips are colored as shown in Fig. S6, except sequences from NCBI whose IDs are shown in black. Gnathostome clades are colored according to protein identity: blue – MANEA (subclades: azure – Clupeocephala except Cypriniformes or Acanthomorpha, cyan – Cypriniformes, melrose: Acanthomorpha), red – MANEAL (subclades: pink – Clupeocephala except Acanthomorpha, dark pink – Acanthomorpha), orange – CMANEAL (subclades: dark yellow – Cypriniformes, light orange – Acanthomorpha). Additional colored clades (from top): deep magenta: diatom GH99 major clades, dark green – rhizarian endosymbiont + chlorophyte GH99 clade, turquoise – rhizarian host GH99 clade, dark violet: jawless vertebrates. The scale shows the expected number of amino acid substitutions per site. Branch labels are UFBoot2 support values. This Supplementary Figure is not intended to be printable.

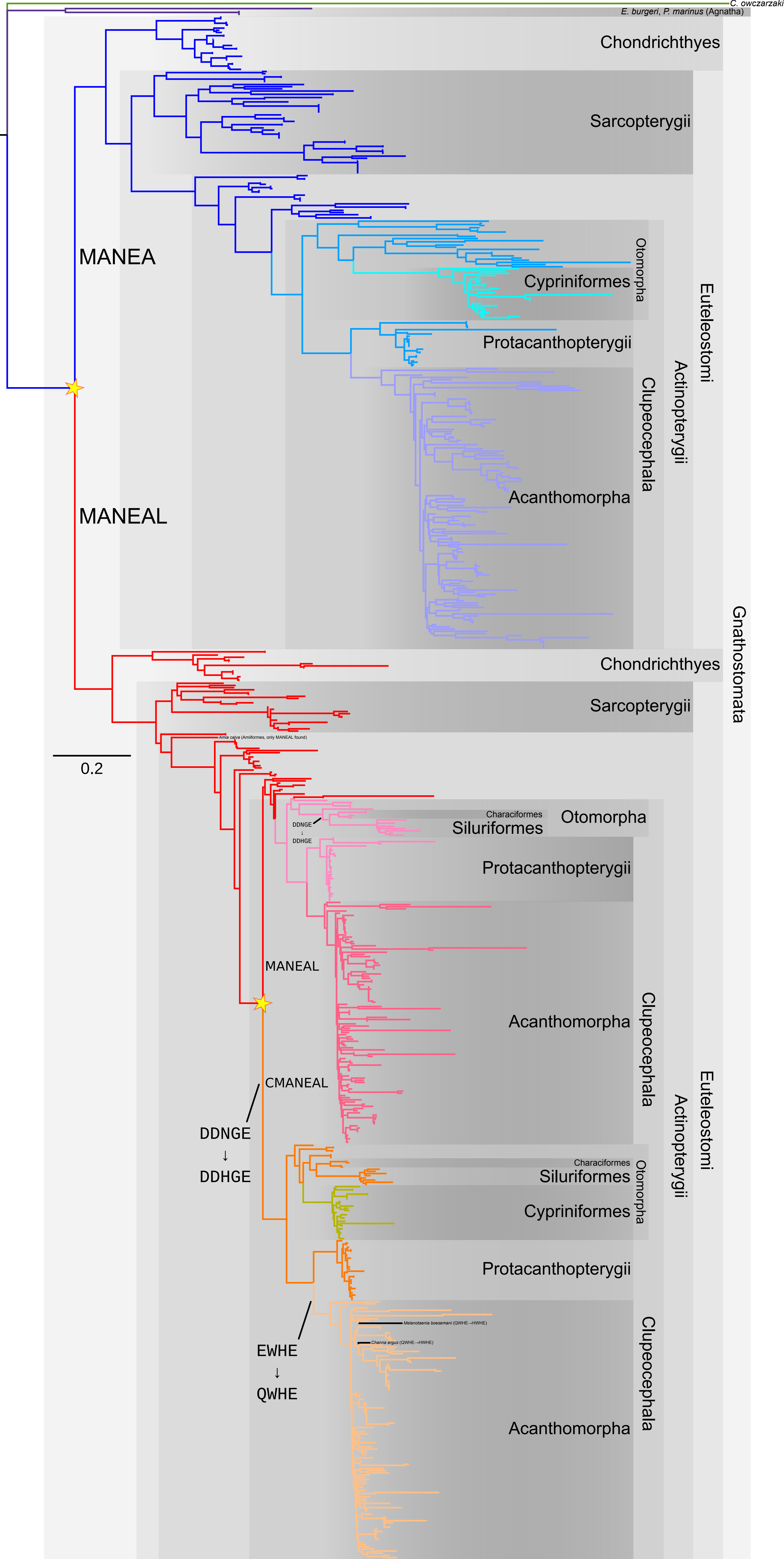

C. owczarzaki
E. burgeri, P. marinus (Agnatha)
Chondrichthyes
Sarcopterygii
MANEA
Otomorpha
Cypriniformes
Protacanthopterygii
Clupeocephala
Acanthomorpha
Actinopterygii
Euteleostomi
Gnathostomata
MANEAL
Chondrichthyes
Sarcopterygii
0.2
Amia calva (Amiiformes, only MANEAL found)
Characiformes
Siluriformes
Otomorpha
DDNGE
↓
DDHGE
Protacanthopterygii
MANEAL
Acanthomorpha
Clupeocephala
Euteleostomi
Actinopterygii
CMANEAL
DDNGE
↓
DDHGE
Characiformes
Siluriformes
Otomorpha
Cypriniformes
Protacanthopterygii
Melanotaenia boesemani (QWHE→HWHE)
Channa argus (QWHE→HWHE)
EWHE
↓
QWHE
Acanthomorpha
Clupeocephala

Supplementary Fig. S2: Annotated phylogeny of vertebrate sequences from LukProt and vertebrate but not tetrapod GH99 sequences found using BLAST of the NCBI nr database. Colors as in Supplementary Fig. S1; the *C. owczarzaki* sequence is used as an outgroup (colored green). Selected evolutionary changes and species are annotated. Stars are gene duplication events. The scale shows the expected number of amino acid substitutions per site.

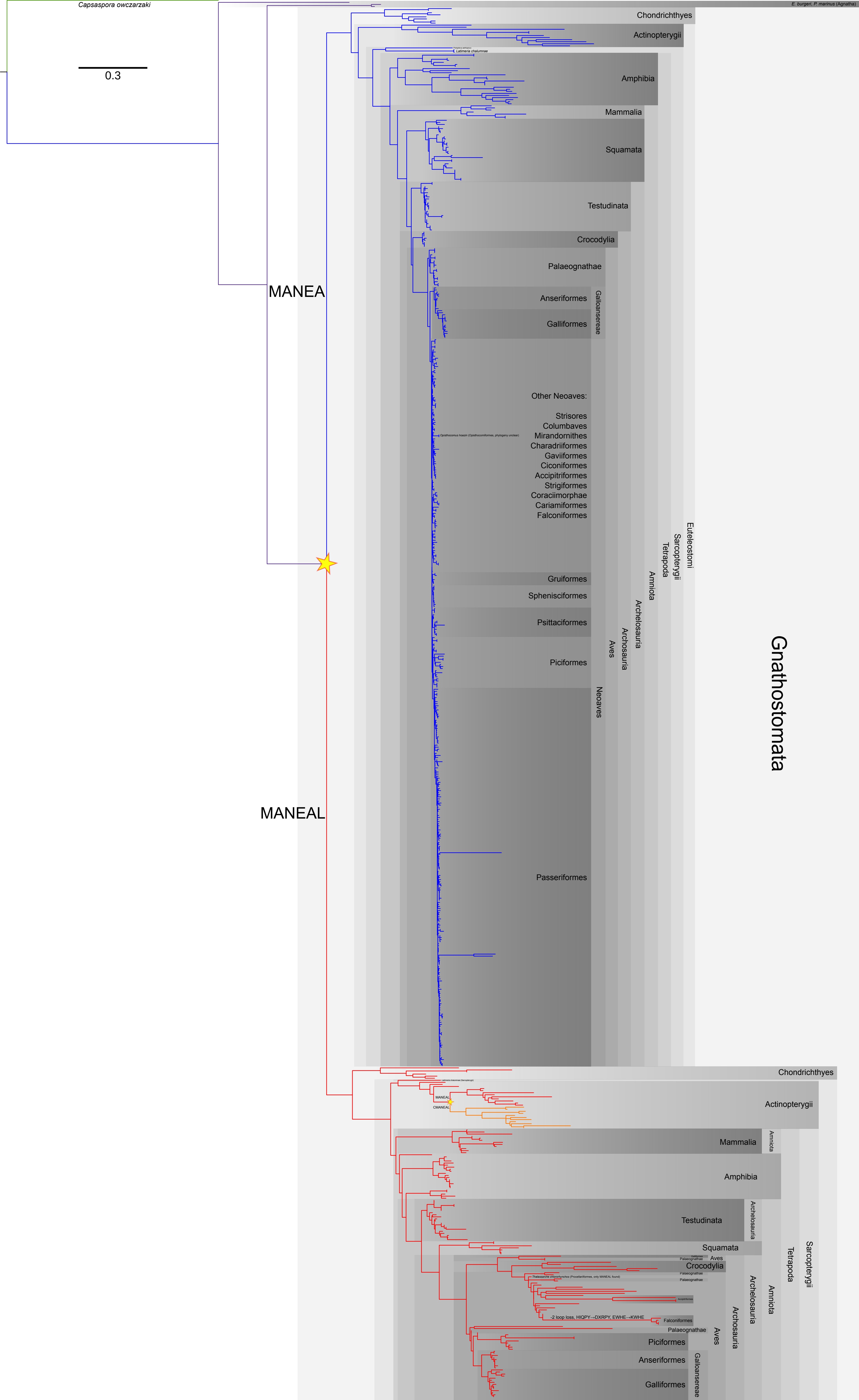

Capsaspora owczarzaki
E. burgeri, P. marinus (Agnatha)
0.3
Chondrichthyes
Actinopterygii
Latimeria chalumnae
Amphibia
Mammalia
Squamata
Testudinata
Crocodylia
Palaeognathae
Anseriformes
Galliformes
Galloansereae
Other Neoaves:
Strisores
Columbaves
Mirandornithes
Charadriiformes
Gaviiformes
Ciconiformes
Accipitriformes
Strigiformes
Coraciimorphae
Cariamiformes
Falconiformes
Opisthocomus hoazin (Opisthocomiformes, phylogeny unclear)
Gruiformes
Sphenisciformes
Psittaciformes
Piciformes
Passeriformes
Neoaves
Aves
Archosauria
Archelosauria
Amniota
Tetrapoda
Sarcopterygii
Euteleostomi
Gnathostomata
MANEA
MANEAL
CMANEAL
Chondrichthyes
Actinopterygii
Mammalia
Amniota
Amphibia
Testudinata
Archelosauria
Squamata
Aves
Crocodylia
Palaeognathae
Thalassarche chlororhynchos (Procellariiformes, only MANEAL found)
-2 loop loss, HIQPY→DXRPY, EWHE→KWHE
Falconiformes
Palaeognathae
Piciformes
Anseriformes
Galliformes
Galloansereae
Aves
Archosauria
Archelosauria
Amniota
Tetrapoda
Sarcopterygii

Supplementary Fig. S3: Annotated phylogeny of vertebrate sequences from LukProt and tetrapod but not mammal GH99 sequences found using BLAST of the NCBI nr database. Colors as in Supplementary Fig. S1 but with no subclade recoloring; the *C. owczarzaki* sequence is used as an outgroup (colored green). Selected evolutionary changes and species are annotated. Stars are gene duplication events. The scale shows the expected number of amino acid substitutions per site.

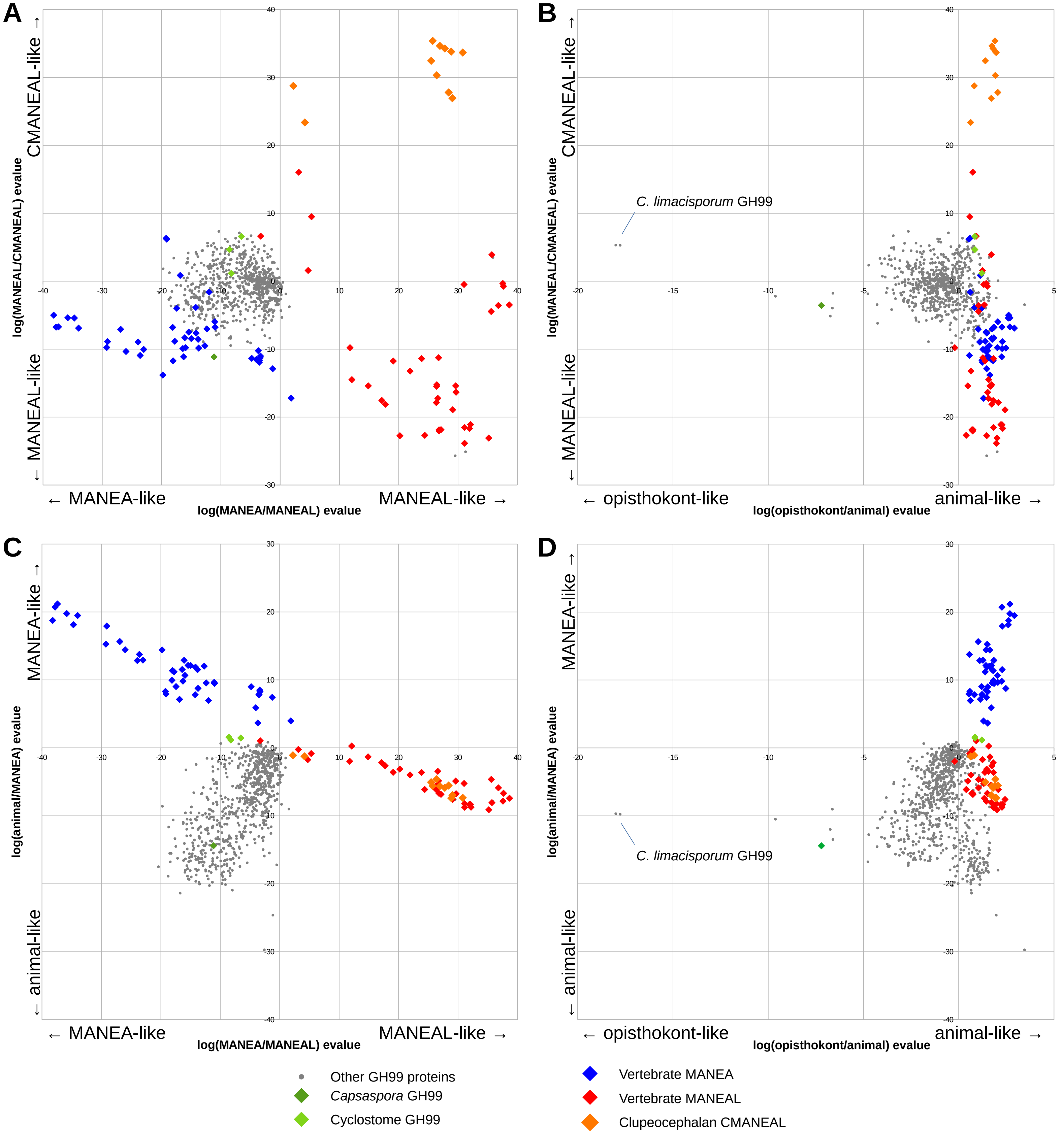
A
B
C
D
CMANEAL-like
MANEAL-like
MANEA-like
animal-like
opisthokont-like
log(MANEAL/CMANEAL) evalue
log(MANEA/MANEAL) evalue
log(opisthokont/animal) evalue
log(animal/MANEA) evalue
C. limacisporum GH99
Other GH99 proteins
Capsaspora GH99
Cyclostome GH99
Vertebrate MANEA
Vertebrate MANEAL
Clupeocephalan CMANEAL

Supplementary Fig. S4: Evaluation of the sensitivity of the five prepared HMM profiles of GH99 proteins, named and prepared from: **opisthokont** GH99, **animal** GH99, vertebrate **MANEA**, vertebrate **MANEAL** and clupeocephalan **CMANEAL**, referred to in bold. Each point represents a single sequence found by searching LukProt using each model and its placement is based on the log($evalue_{model\ 1}$/$evalue_{model\ 2}$). E-value is a measure of the probability of a hit being a false positive, it is lower for sequences matching a profile and higher for sequences less similar to a profile. The more negative the value of log($evalue_{model\ 1}$/$evalue_{model\ 2}$), the more likely the sequence is to be found by model 1. The more positive this value, the more likely it is to be found by model 2. These values are treated here as dimensions. Selected dots are colored according to the protein they represent (see the Figure legend). The axes were additionally labeled with arrows to make interpretation easier. The plots were composed so that adjacent, parallel axes are the same. (A) log(**MANEAL**/**CMANEAL**)$_{evalue}$ (Y axis) versus log(**MANEA**/**MANEAL**)$_{evalue}$ (X axis). The three ohnologs separate to the expected parts of the plot, with the evalue of true CMANEAL hits found by the **CMANEAL** profile being always lower than for the same sequences found by the **MANEAL** profile. (B) log(**MANEAL**/**CMANEAL**)$_{evalue}$ (Y axis) versus log(**opisthokont**/**animal**)$_{evalue}$ (X axis) . Here one can see that neither the **opisthokont** profile, nor the **animal** profile can differentiate between MANEA, MANEAL or CMANEAL, but animal sequences are in general preferentially found by the **animal** profile. (C) log(**animal**/**MANEA**)$_{evalue}$ (Y axis) versus log(**MANEA**/**MANEAL**)$_{evalue}$ (X axis). Most GH99 protein sequences are more MANEA-like than MANEAL-like, signifying that MANEAL and CMANEAL diverged from the ancestral sequences more than MANEA did. The **MANEA** profile is more sensitive towards MANEA proteins than the **animal** profile. (D) log(**animal**/**MANEA**)$_{evalue}$ (Y axis) versus log(**opisthokont**/**animal**)$_{evalue}$ (X axis). Again, the **MANEA** profile is more sensitive towards MANEA proteins than others, and the **animal** profile is more sensitive towards the animal proteins than the **opisthokont** profile. GH99 sequences from known genes were colored according to the legend. The profiles are supplied in the associated Zenodo repository.

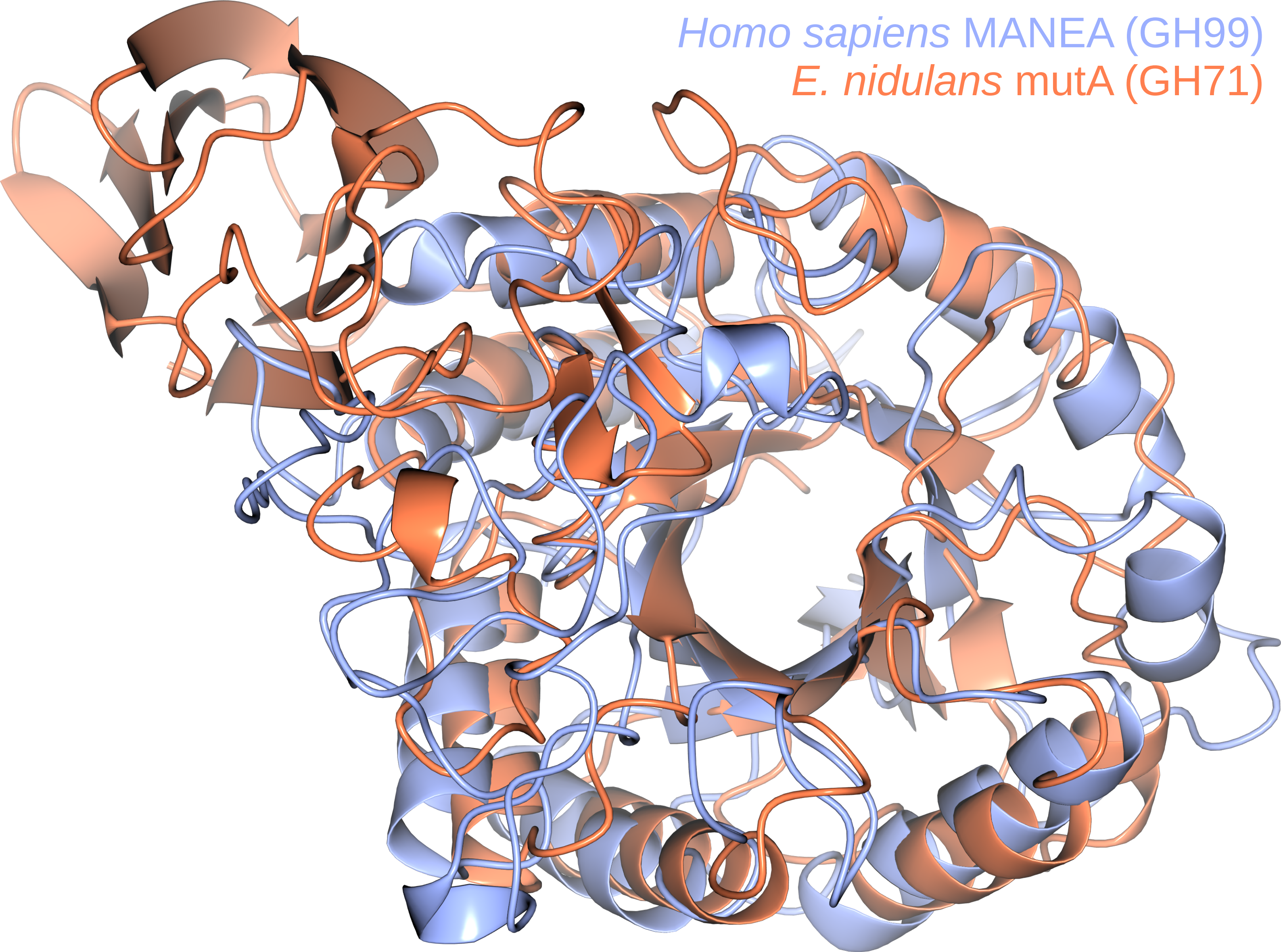
Homo sapiens MANEA (GH99)
E. nidulans mutA (GH71)

Supplementary Fig. S5: 3D alignment of human MANEA structure (ice blue, PDB ID: **6ZFA**) and the AlphaFold model of *E. nidulans* GH71 mutanase (gold, UniProt ID: **Q96VT3**). Structures were aligned in ccp4mg using main chain atoms of residues 404-407 in **6ZFA** and of residues 265-268 in **Q96VT3** AphaFold model v4.

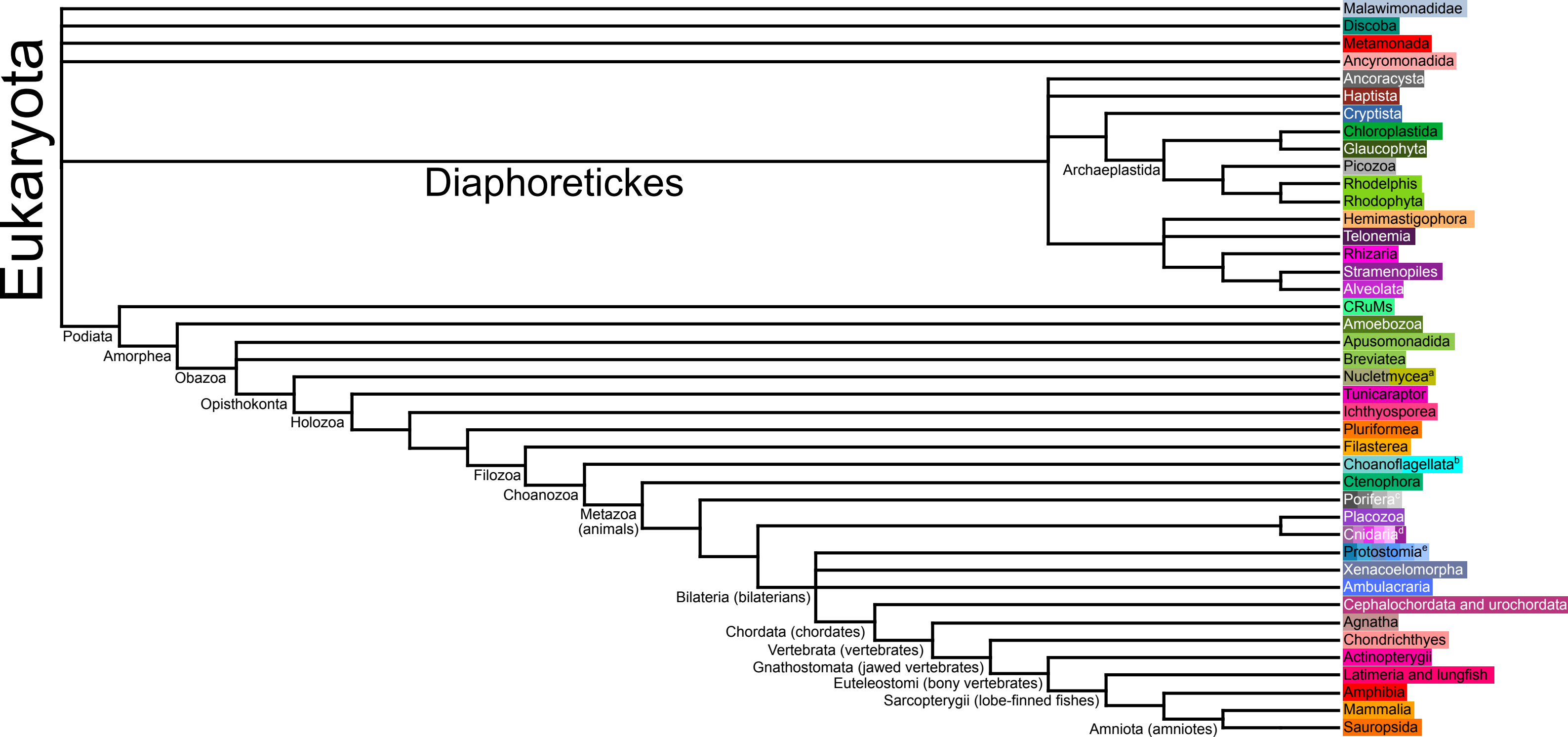

Eukaryota
Diaphoretickes
Malawimonadidae
Discoba
Metamonada
Ancyromonadida
Ancoracysta
Haptista
Cryptista
Chloroplastida
Glaucophyta
Picozoa
Rhodelphis
Rhodophyta
Archaeplastida
Hemimastigophora
Telonemia
Rhizaria
Stramenopiles
Alveolata
CRuMs
Amoebozoa
Apusomonadida
Breviatea
Nucletmycea[a]
Tunicaraptor
Ichthyosporea
Pluriformea
Filasterea
Choanoflagellata[b]
Ctenophora
Porifera[c]
Placozoa
Cnidaria[d]
Protostomia[e]
Xenacoelomorpha
Ambulacraria
Cephalochordata and urochordata
Agnatha
Chondrichthyes
Actinopterygii
Latimeria and lungfish
Amphibia
Mammalia
Sauropsida
Podiata
Amorphea
Obazoa
Opisthokonta
Holozoa
Filozoa
Choanozoa
Metazoa (animals)
Bilateria (bilaterians)
Chordata (chordates)
Vertebrata (vertebrates)
Gnathostomata (jawed vertebrates)
Euteleostomi (bony vertebrates)
Sarcopterygii (lobe-finned fishes)
Amniota (amniotes)

Supplementary Fig. S6: Phylogeny tip coloring scheme used in all trees. Clade names are written under the corresponding branches. The following clades have multiple colors in their subclades (listed from left to right): [a]Nucletmycea: Rotosphaerida, Fungi; [b]Choanoflagellata: Craspedida, Acanthoecida; [c]Porifera: Demospongiae, Hexactinellida, Homoscleromorpha, Calcarea; [d]Cnidaria: Cubozoa, Hydrozoa, Scyphozoa, Hexacorallia, Octocorallia, Myxozoa; [e]Protostomia: Nematoda, Arthropoda, Platyhelminthes, Mollusca, Annelida, other Lophotrochozoa.

Descriptions of Supplementary Tables

Supplementary Table SI: Data on further investigation of taxa not covered by the LukProt database but essential for finding gains and losses of the endomannosidase by various clades, categorized by taxogroup. These investigations were done mostly using genomic TBLASTN. The search methodology and the result are listed in every case.

Supplementary Table SII: Chromosomal loci of GH99 genes in various vertebrates. Column A refers to the species investigated and columns B and C are the chromosomal positions of, respectively, *MANEA* and *MANEAL* genes. The name of the assembly used for the analysis is in column D. CLGK refers to the protovertebrate Conserved Linkage Group K (see main text).

Supplementary Table SIII: List of the accession numbers of MANEA protein sequences misclassified as MANEAL in public databases.

Supplementary Table SIV: Table of sequence identifiers of GH99 sequences, corresponding species and taxogroups and protein classification (MANEA/MANEAL/CMANEAL), as well as sequence cleaning status (retained, divergent or contamination).

Supplementary Table SV: A table summarizing phylogeny-based sequence cleaning rounds. In column A the sequence ID is shown. Column B states the reason for sequence removal (extreme divergence which causes phylogeny artifacts or predicted contamination – resulting from source sequence contamination by other species). In column C, the round at which a given sequence was removed is shown; after each round, a new phylogeny was calculated and reassessed, this was done until no such sequences were found.

Supplementary Table SVI: Abundance of sequences and particular sequence features in GH99 proteins from various eukaryotic taxogroups. Abbreviations are explained in the sheet “abbreviation explanations”.

Supplementary Table SVII: Lists of species from various taxogroups categorized by the proteins they harbor. Species highlighted blue were found in public databases to only have MANEA, species highlighted red were thought to only have MANEAL and non-highlighted species were found in databases to contain both proteins. Species were moved to appropriate columns after classification performed in this work but their highlighting was not changed to show errors currently existing in public databases.
